# Singular observation of the polarization-conversion effect for a gammadion-shaped metasurface

**DOI:** 10.1038/srep22196

**Published:** 2016-02-26

**Authors:** Chu-En Lin, Ta-Jen Yen, Chih-Jen Yu, Cheng-Min Hsieh, Min-Han Lee, Chii-Chang Chen, Cheng-Wei Chang

**Affiliations:** 1 Department of Mechanical Engineering, National Chin-Yi University of Technology, Taichung 411, Taiwan; 2 Department of Material Science and Engineering, National Tsing Hua University 300 Hsinchu, Taiwan; 3 Graduate Institute of Electro-Optical Engineering, Chang Gung University 333 Taoyuan, Taiwan; 4 Department of Optics and Photonics, National Central University 320 Taoyuan, Taiwan

## Abstract

In this article, the polarization-conversion effects of a gammadion-shaped metasurface in transmission and reflection modes are discussed. In our experiment, the polarization-conversion effect of a gammadion-shaped metasurface is investigated because of the contribution of the phase and amplitude anisotropies. According to our experimental and simulated results, the polarization property of the first-order transmitted diffraction is dominated by linear anisotropy and has weak depolarization; the first-order reflected diffraction exhibits both linear and circular anisotropies and has stronger depolarization than the transmission mode. These results are different from previously published research. The Mueller matrix ellipsometer and polar decomposition method will aid in the investigation of the polarization properties of other nanostructures.

In recent years, there have been demands for miniature optical devices that are thinner, lighter and more efficient^1^,^2^,^3^. Thus, some chiral nanostructures were proposed for significant phenomena in polarization conversion. Gammadion-shaped metasurfaces have been discussed extensively because they are potential devices for optical applications. According to the previous research, the polarization conversion is caused by circular birefringence (CB) because of the asymmetric structures^4^,^5^. In 2003, Papakostas *et al.* proposed an asymmetric gammadion-shaped structure that could rotate incident linearly polarized light at an angle. They claimed that the phenomenon resulted from optical activity^5^. In 2012, a review article by Li *et al.* also concluded that some asymmetric nanostructures could rotate the incident polarization state with CB^6^. However, the phenomenon of optical rotation results from CB, linear birefringence (LB), and linear diattenuation (LD), for example, LB, such as a half-wave plate, CB, such as a quartz crystal or glucose water solution, and linear diattenuation, such as a linear polarizer, can change the azimuth angle of incident polarized light^7^,^8^,^9^. Additionally, the change in the ellipticity angle results from the contributions of circular diattenuation (CD) and LB. A polarimetric method that completely characterizes the polarization conversion mechanism of the gammadion-shaped metasurfaces will aid our understanding of the mechanism of the optical-rotation phenomena. To clearly describe the polarization transfer function of the gammadion-shaped metasurface, in 1943, Hans Mueller proposed the idea of Mueller calculus to study the relationships among the polarization effect of input light, output light, and materials^10^. Thus, the Mueller matrix can be used to determine the polarization properties of a material and predict the polarization state of the output light. Furthermore, Owing to the measured Mueller matrix, this matrix can be decomposed to analyze the three polarization properties of depolarization, diattenuation, and retardation according to the polar decomposition^11^,^12^,^13^. Unlike the well-known Jones calculus^14^, Mueller calculus provides more information, such as unpolarized light, partially polarized light, and depolarization.

In this study, the Mueller matrix ellipsometer was used to investigate and analyze the polarization-conversion mechanism of gammadion-shaped nanostructures. The polarization-conversion effect did no result from CB only, which is in contrast to previous research^5^,^6^. The experimental outcome indicates that the optical-rotation phenomenon of transmitted light of first-order diffraction is dominated by LD and that the change of ellipticity angle is dominated by LB. Additionally, for the reflection case, the optical-rotation phenomenon of reflected light of first-order diffraction is contributed by LD and CB, and the change of ellipticity angle is caused by CD and LB. Through this study, the weighting of LB, CB, LD, and CD is demonstrated.

## Results

Three types of gammadion-shaped metasurfaces (GMSs) with different branches and metal thicknesses are fabricated and simulated. The objective of modifying these physical parameters of the GMS (number of branches and metal thickness) is to investigate the influence of these parameters on the type of optical anisotropy produced. The physical parameters of the fabricated gammadion-shaped nanostructures are shown in Table 1 in which the linewidth, the metal thickness, and the number of branches are considered.

**Figure 1:**
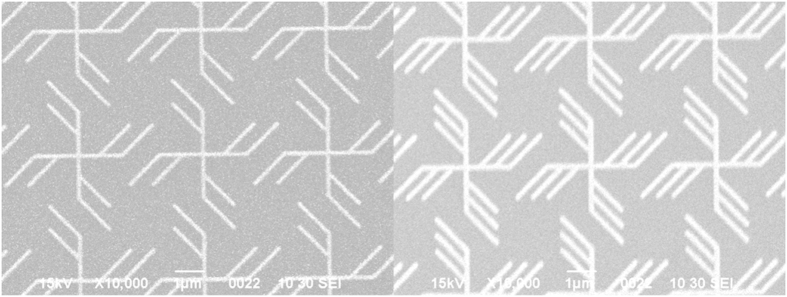
(**a**) SEM image of the two-branch structure. (**b**) SEM image of the three-branch structure.

The scanning electron microscopy (SEM) image of the two-branch gammadion-shaped nanostructures G215 and the three-branch G315 are illustrated in Fig. 1(a,b), respectively.

**Figure 2:**
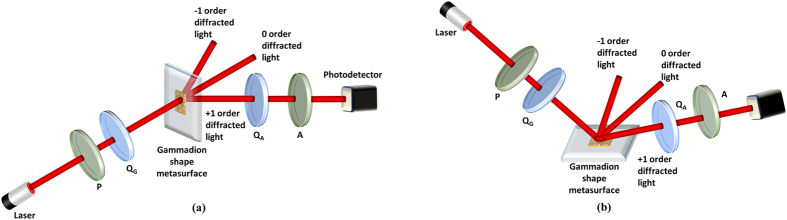
Experimental setup of the Mueller matrix ellipsometer, where P and A are polarizers and Q_G_ and Q_A_ are quarter-wave retarders. (**a**) Optical setup for measuring the polarization properties of the first-order transmitted diffraction of the nanostructure with gammadion shape. (**b**) Optical setup for measuring the polarization properties of the first-order reflected diffraction of the nanostructure with gammadion shape.

For the experimental arrangement, we used a Mueller matrix ellipsometer to study the polarization properties of the gammadion-shaped nanostructure. A frequency-stabilized He-Ne laser (R-32734 Newport, Irvine, CA, United States) was used as the light source for the Mueller matrix ellipsometer, whose central wavelength at 632.8 nm was incident on the sample (Fig. 2) in the transmission mode and reflection mode.

The polarization transfer function of the first-order transmitted diffraction under the normal incidence describes the transmission properties of the gammadion-shaped metasurface. Additionally, the reflection properties are used to characterize the first-order reflected diffraction light with oblique incidence at an incident angle of 70°. The measured Mueller matrix (shown in the supplementary information) is decomposed into three Mueller matrices using polar decomposition^11^. Furthermore, the polarization properties, including LD, CD, LB, CB, and depolarization, are calculated from the decomposed Mueller matrices. The results for transmission and reflection modes are listed in Tables 2 and 3.

According to the measured result from Table 2, for the transmission mode, all tested samples are weak-depolarizing (see row Δ). For 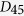, the nanostructures express the strongest diattenuation in the +45°/−45° direction. However, the horizontal diattenuation 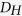 and circular diattenuation 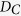 are at least fivefold weaker than 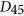. Thus, the effect from 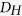 and 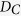 could be neglected. For retardation (see the rows of linear phase retardation Γ, fast axis orientation *ψ*, and circular phase retardation Φ), the fast axis of the linear phase retarder is close to −45°, and the circular phase retardation is close to zero. As a result, when the incident light is normal-incident upon a gammadion-shaped structure, the polarization conversion from the incident light to the first diffracted light exhibits linear-amplitude and linear-phase anisotropies, and the principal axes of the linear-amplitude and linear-phase anisotropies nearly coincide.

For the reflection mode, shown in Table 3, the polarization properties of the first-order reflected diffraction of the measured gammadion metasurface exhibit the following characteristics: (1) the depolarization is stronger than that of the transmitted mode. (2) The linear anisotropy is stronger than the circular anisotropy, which implies that Γ is larger than Φ, and the linear diattenuation, defined as 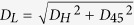, is also either nearly equal to or larger than the circular diattenuation 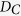.

**Figure 3:**
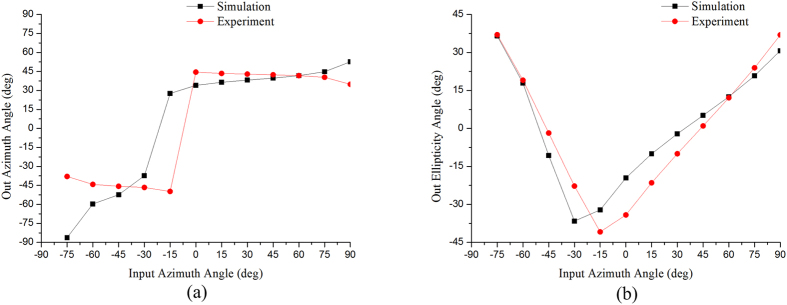
(**a**) Azimuth angle and (**b**) ellipticity angle of the output polarization state of the first-order diffracted light transmitted from the G315 nanostructure with a gammadion shape under different input linear polarization states. The red dots are the experimental results, and the black dots are the simulated results.

**Figure 4:**
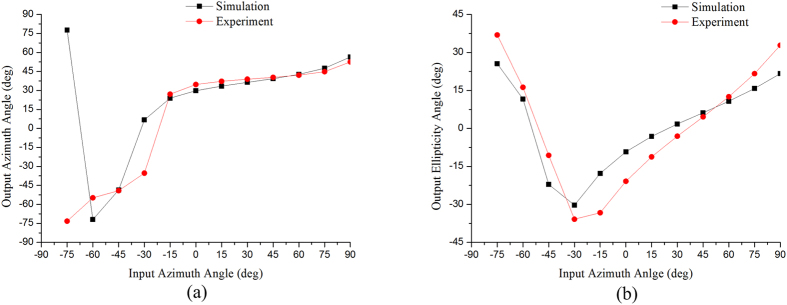
(**a**) Azimuth angle and (**b**) ellipticity angle of the output polarization state of the first-order diffracted light transmitted from the G215 nanostructure with a gammadion shape under different input linear polarization states. The red dots are the experimental results, and the black dots are the simulated results.

**Figure 5:**
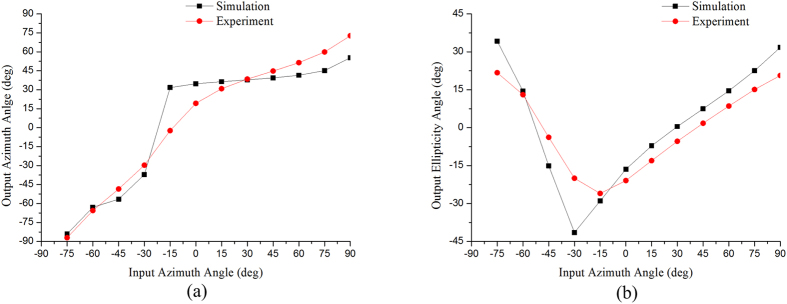
(**a**) Azimuth angle and (**b**) ellipticity angle of the output polarization state of the first-order diffracted light transmitted from the G218 nanostructure with a gammadion shape under different input linear polarization states. The red dots are the experimental results, and the black dots are the simulated results.

A computer simulation is also used to predict the emergent polarization state of the first-order diffracted light transmitted from the gammadion-shaped metasurface when a series of linearly polarized light with different azimuth angles is incident upon the surface. In Figs 3, 4 and 5, the red dots are the results simulated by CST software (Computer Simulation Technology, Framingham, MA, United States), and the black dots are the experimental results, which are obtained by the following steps. An experimental Stokes vector of the emergent polarization state is obtained by multiplying the experimental Mueller matrix of the tested specimen with an ideal incident polarization state; thus, the azimuth angle *θ* and ellipticity angle *ε* are obtained from equations (1) and (2):


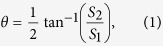


and


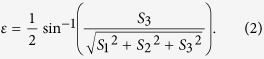


where 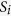 (*i* = 0, 1, 2, 3) are the elements of the Stokes vector. A comparison of the experimental results with the simulated results indicates that our experimental results correspond well with the theoretical predictions. In accordance with Table 2, the principal axes of the measured metasurfaces are close to +45°/−45°. In other words, we can deduce the eigen-polarization states of the gammadion-shaped metasurface are +45° and −45° linearly polarized light (see Figs 3, 4, 5). This investigation provides strong evidence that the eigen-polarization state is a pair of the mutually and nearly linear polarization states. Because the polarization property of the first-order transmitted diffraction of these metasurfaces is similar to purely linear anisotropy, the azimuth angle change results from the linear amplitude anisotropy (LD) and the ellipticity angle change results from the linear phase anisotropy (LB). This observation is in contrast to previously proposed explanations.

## Discussion

The measured results indicate that the optical rotation induced by an artificial chiral nanostructure is not simply a CB component. In our experiment, the contribution of the polarization-conversion effect from the circular phase and the amplitude anisotropies is lower than found in previous research. By using the Mueller matrix ellipsometer, one can investigate the polarization properties (depolarization, diattenuation, and retardation) through the output light that passes through an undetermined material. In this study, the polarization properties of the first-order transmitted diffraction of a gammadion-shaped metasurface are dominated by linear anisotropy and have weak depolarization. Moreover, the polarization properties of the first-order reflected diffraction of a gammadion-shaped metasurface exhibits both linear and circular anisotropies and has stronger depolarization.

The contribution of the linear anisotropy and circular anisotropy of the polarization properties in the first- order transmitted diffraction and first-order reflected diffraction of the GMS might result from the geometry of the gammadion-shaped nanostructure. In transmission mode, we conclude that the polarization rotation effect results from the linear diattenuation, and the change in the ellipticity results from the linear phase retardation. In reflection mode, the polarization rotation effect results from the synergy of linear diattenuation and circular birefringence, and the change in the ellipticity results from the synergy of the circular diattenuation and linear birefringence.

This gammadion-shaped structure can be decomposed into two parts: the cross and the branches. The induced optical anisotropy of the GMS might be explained according to the research on V-shaped nanostructures^15^,^16^,^17^, in first-order transmitted diffraction, the branches and the cross can be treated as the V-shaped nanostructure with different included angles. We could deduce that the linear phase retardation results from the parts of the included angle between the branches and the cross. According to the articles published by Yu *et al.*^15^, the included angle of the V-shaped nanostructure is related to the linear phase retardation. They discussed the reason for linear phase retardation using generalized laws of reflection and refraction derived from Fermat’s principle^15^. Thus, the angle between the branch and the cross results in the change in the ellipticity. Moreover, the metal branches and cross could contribute to the linear diattenuation, which could result in the polarization rotation. Thus, the polarization effect of the metal branches and cross could be recognized as a metal grid. Similar to the wire-grid partial polarizer^18^, the cross portion of the metal layer generates the surface current along the x- and y-directions equally so that the net effect is the induction of the 45°-linear diattenuation. The branches are oriented at 45° and −45°, and they contribute to the linear diattenuation.

**Figure 6:**
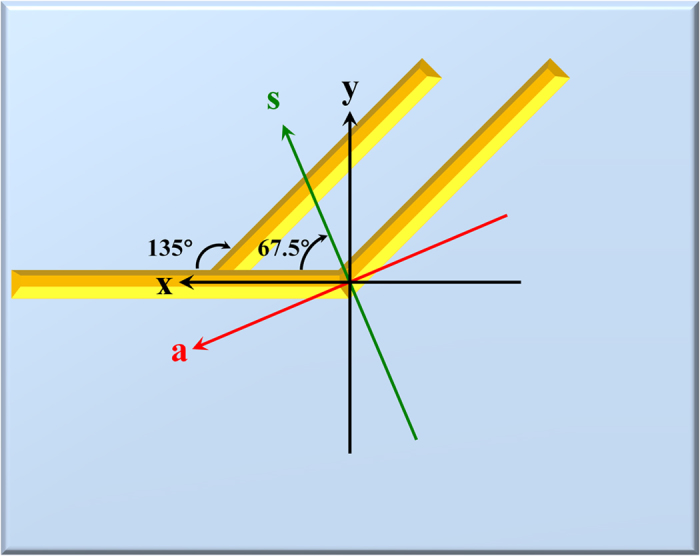
The symmetry axis (**s**) and asymmetry axis (**a**) of the branch of the gammadion-shaped metasurface.

In first-order reflected diffraction, the linear anisotropy might result from the contribution of the branches. The branches can be treat as a V-shape nanostructure, in theory, the symmetry axis of the branches of the designed GMS is equal to 67.5° (Fig. 6), which is in contrast to the experimental results in which the fast axis is close to the symmetry axis of the branch as well as the V-shaped nanostructure. Additionally, circular anisotropy is only observed in the reflection but not in the transmission. This is because the oblique incidence has in plane and out of plane polarized components; however, the normal incidence only possesses the in plane polarized component. We suggest that the in plane excitation may contribute to the linear anisotropy, while the out of plane may contribute to the circular anisotropy.

In the near future, we will use the Mueller matrix ellipsometer and the polar-decomposition method to study the polarization properties of other nanostructures.

## Methods

### Preparation of the gammadion-shaped metasurface

An E-beam writer (Elionix, ELS-7800) is used to draw the gammadion-shaped pattern. Indium tin oxide (ITO) is deposited with a thickness of 3 nm on a glass slide (1 cm × 1 cm). Then, a photoresist spinner (MS-A100, Mikasa, Tokyo, Japan) is used to coat photoresist (PMMA950) on the substrate, which is placed on a hot plate (HP-11 LA, Askul, Tokyo, Japan) for gentle heating for 90 s at 200 °C. After the E-beam process, the slide is immersed in a 1:3 methyl isobutyl ketone (MIBK): isopropyl alcohol (IPA) developer solution for 1 min and then in IPA for 20 s. To deposit the metal onto the glass, we placed the glass slide in an E-gun evaporator (Fulintec, FU-PEB-500). First, we deposited a Ti thin film with a thickness of 5 nm as an adhesive layer, and then, we deposited gold with a thickness of 50 nm and 80 nm. Note that the deposition rate is controlled at 0.5 Å/s. In the lift-off process, we placed the deposited glass slide in acetone and gently agitated the beaker. When the gold film on the photoresist was lifted off, the sample preparation for measurement was complete.

### Computer simulation

To simulate the gammadion-shaped metasurfaces, we used commercial finite element-based electromagnetic field solver (CST Microwave StudioTM) and calculated the polarization rotation and ellipticity in first order transmitted diffraction light. Two types of complex gammadion-shaped structures were designed and simulated. The bottom substrate is porous silica, and a 5-nm thick indium tin oxide (ITO) layer lies on top of the silica. The corrugated metallic gammadion arrays consist of 5 nm Ti as the adhesive layer and 50 nm and 80 nm gold film. Then, these gammadion-shaped metasurfaces were illuminated by a linearly polarized plane wave with a wavelength of 632.8 nm and with a periodic boundary condition. The azimuth angle of the incident linear polarization state was varied from 0° to 180°, and the interval of the simulated point was 15°. The refractive index of the glass is 1.5, and the material properties of ITO, Ti and Au are based on ref. [19], ref. [20] and ref. [20], respectively. In the simulation, the polarization rotation azimuth angle and the ellipticity angle were calculated using the Stokes parameter for different types of structures.

### Characterization of the polarization properties of a gammadion-shaped metasurface

Theoretically, the ensemble polarization conversion from a photonic device or material is caused by optical anisotropy. Optical anisotropy includes linear amplitude anisotropy, linear phase anisotropy, circular amplitude anisotropy, and circular phase anisotropy. The polarization transfer function directly reflects the polarization characteristics for an unknown optical system or the optical components. In this study, we analyze the amplitude and phase anisotropies of the gammadion-shaped nanostructure in terms of the Mueller calculus. The relation between light and the optical system can be represented as 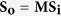 or


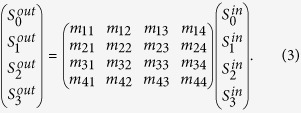


where **S**_**o**_ and **S**_**i**_ are the Stokes vectors of the output and input light, respectively, and **M** is the Mueller matrix for an arbitrary optical system. According to the method proposed by Lu and Chipman, the Mueller matrix of an undetermined specimen can be divided into three matrices^11^:


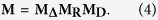


where 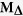, 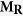, and 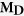 are the Mueller matrices for depolarization, phase retardation, and diattenuation, respectively. Matrix M can be decomposed into these three matrices according to polar decomposition, which is described in ref. 10 in detail. In equation (4), 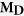 describes the polarization-dependent transmission of the undetermined specimen. The polarization properties of a diattenuator can be characterized by three diattenuations, which are the horizontal diattenuation, 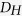, 45°-linear diattenuation, 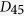, and circular diattenuation, 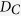, defined as


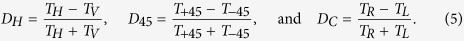


where *T* is the transmittance and the subscripts H, V, +45, −45, R, and L denote horizontally, vertically, +45° linearly, −45° linearly, right-handed circularly, and left-handed circularly polarized light, respectively. These three diattenuations indicate the amplitude anisotropy of the specimen. 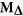 describes the capability of depolarization by the undetermined specimen, which can depolarize the incident polarization state. Furthermore, the diagonal matrix elements of 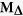: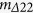, 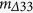, and 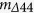 indicate the depolarization coefficients for depolarizing the incident horizontal, 45° linear, and circular polarization states, respectively. The net depolarization power of the undetermined specimen is determined by the parameter Δ, which can be obtained from


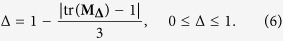


where 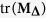 is the trace of 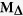. A specimen is completely depolarizing when Δ approaches 1. In theory, 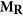 can be further decomposed into a linear phase retarder 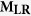 and a circular phase retarder 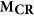. 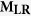 would follow 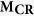, such as 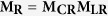. For 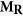, the corresponding parameters are Γ, *ψ*, and Φ, which represent the linear phase retardation, fast-axis angle, and circular phase retardation, respectively. These three parameters can express the phase anisotropy. Theoretically, once 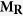 is obtained, the parameters Γ, *ψ*, and Φ can be calculated from equations (7), (8), and (9), respectively.


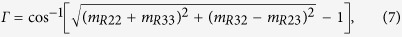



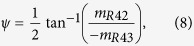


and


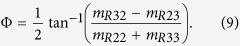


where 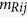 (*i*, *j* = 1, 2, 3, 4) are the Mueller matrix elements of 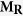.

### Measurement of the Mueller matrix ellipsometer

Mueller matrix ellipsometry and Fourier analysis are combined to measure the 15 normalized Mueller matrix elements of a gammadion-shaped metasurface (normalized by 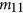; see eq. (3))^21^. We utilize the Mueller matrix ellipsometer illustrated in Fig. 2(a,b) to measure the Mueller matrix of the transmission and reflection properties of the gammadion-shaped metasurface, respectively. In Fig. 2, P and A are the polarizer and analyzer, and their transmission axes are preset to be parallel to the x-axis. Q_G_ and Q_A_ are the quarter-wave retarders whose fast axes are also preset to be parallel to the x-axis. Then, Q_G_ and Q_A_ rotate simultaneously by the motorized rotation stages, and the ratio of the angular speeds is 5:1^10^. Thus, we have


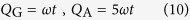


The time-varying intensity signal 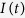 is received by the photodetector, and this signal has fundamental and harmonic components^10^. According to Fourier theory, we can decompose 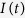 into cosine series and sine series, such as


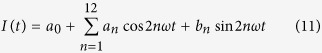


According to equation (11), the Fourier amplitudes 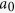, 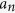, and 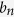 (*n* = 1, 2, 3,…) can be obtained by Fourier analysis. These Fourier amplitudes are a function of the Mueller matrix elements 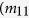, 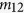, …, 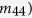, which is described in Table 1 of ref. 10. Thus, we can obtain the Mueller matrix elements from the Mueller matrix ellipsometer.

## Additional Information

**How to cite this article**: Lin, C.-E. *et al.* Singular observation of the polarization-conversion effect for a gammadion-shaped metasurface. *Sci. Rep.* **6**, 22196; doi: 10.1038/srep22196 (2016).

## Supplementary Material

Supplementary Information

## Figures and Tables

**Table 1:**
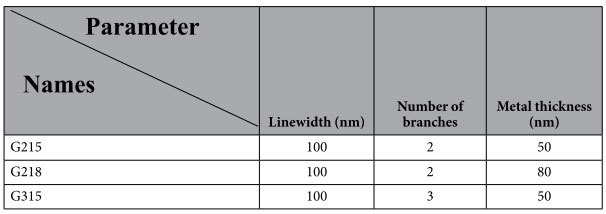
Physical parameters of the fabricated samples.

**Table 2:** Polarization properties of the gammadion-shaped nanostructures under the transmission measurements.

	First-order transmitted diffraction		
	G315	G215	G218
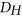	0.041 ± 0.000	0.119 ± 0.002	0.005 ± 0.002
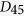	0.338 ± 0.001	0.601 ± 0.005	0.416 ± 0.002
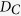	0.055 ± 0.002	−0.008 ± 0.001	−0.027 ± 0.003
Φ (deg)	0.348 ± 0.055	−0.641 ± 0.059	0.623 ± 0.141
Γ (deg)	91.934 ± 0.282	78.133 ± 0.188	48.503 ± 0.277
*ψ* (deg)	−46.350 ± 0.035	−51.951 ± 0.064	−47.995 ± 0.140
Δ	0.026 ± 0.002	0.014 ± 0.006	0.066 ± 0.003

**Table 3:** Polarization properties of the gammadion-shaped nanostructures under the reflection measurements.

	First-order reflected diffraction		
	G315	G215	G218
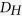	0.157 ± 0.021	0.360 ± 0.012	0.059 ± 0.006
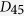	−0.137 ± 0.013	−0.015 ± 0.001	−0.086 ± 0.007
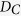	−0.241 ± 0.003	−0.013 ± 0.007	−0.011 ± 0.001
Φ (deg)	−27.795 ± 0.755	−46.500 ± 0.448	−62.903 ± 0.517
Γ (deg)	73.278 ± 0.982	76.522 ± 0.326	109.796 ± 0.148
*ψ* (deg)	63.226 ± 0.307	62.961 ± 0.312	59.561 ± 0.203
Δ	0.245 ± 0.006	0.285 ± 0.005	0.166 ± 0.001
