# Supplementary material for: Singular observation of the polarization-conversion effect for a gammadion-shaped metasurface
Source: Sci Rep. 2016 Feb 26;6:22196. doi: 10.1038/srep22196 (PMC4768175; doi:10.1038/srep22196)
Supplement: Supplementary Information [file srep22196-s1.pdf]

Supplementary Information for Singular Observation of the Polarization-Conversion Effect for a Gammadion-Shaped Metasurface by Chu-En Lin, Ta-Jen Yen, Chih-Jen Yu, Cheng-Min Hsieh, Min-Han Lee, Chii-Chang Chen, and Cheng-Wei Chang

The experimental Mueller matrices of the gammadion-shaped metasurfaces

G315 first-order transmitted diffraction

Experimental Mueller matrix

$$\mathbf{M}=\begin{pmatrix} 1.000\pm0.000 & 0.041\pm0.000 & 0.338\pm0.001 & 0.055\pm0.002 \\ 0.050\pm0.004 & -0.043\pm0.006 & 0.066\pm0.004 & 0.894\pm0.006 \\ 0.311\pm0.002 & 0.058\pm0.003 & 0.961\pm0.004 & -0.040\pm0.001 \\ 0.001\pm0.001 & -0.933\pm0.002 & 0.043\pm0.001 & -0.014\pm0.003 \end{pmatrix}$$

$$\mathbf{M_D}=\begin{pmatrix} 1.000\pm0.000 & 0.041\pm0.000 & 0.338\pm0.001 & 0.055\pm0.002 \\ 0.041\pm0.000 & 0.939\pm0.000 & 0.007\pm0.000 & 0.001\pm0.000 \\ 0.338\pm0.001 & 0.007\pm0.000 & 0.998\pm0.000 & 0.010\pm0.000 \\ 0.055\pm0.002 & 0.001\pm0.000 & 0.010\pm0.000 & 0.940\pm0.000 \end{pmatrix}$$

$$\mathbf{M_R}=\begin{pmatrix} 1.000\pm0.000 & 0.000\pm0.000 & 0.000\pm0.000 & 0.000\pm0.000 \\ 0.000\pm0.000 & -0.031\pm0.005 & 0.061\pm0.001 & 0.998\pm0.000 \\ 0.000\pm0.000 & 0.049\pm0.001 & 0.997\pm0.000 & -0.059\pm0.001 \\ 0.000\pm0.000 & -0.998\pm0.000 & 0.047\pm0.001 & -0.034\pm0.005 \end{pmatrix}$$

$$\mathbf{M_\Delta}=\begin{pmatrix} 1.000\pm0.000 & 0.000\pm0.000 & 0.000\pm0.000 & 0.000\pm0.000 \\ -0.022\pm0.005 & 0.955\pm0.006 & 0.006\pm0.002 & 0.018\pm0.002 \\ -0.016\pm0.004 & 0.006\pm0.002 & 0.971\pm0.006 & -0.008\pm0.002 \\ 0.030\pm0.002 & 0.018\pm0.002 & -0.008\pm0.002 & 0.995\pm0.001 \end{pmatrix}$$

G215 first-order transmitted diffraction

$$\mathbf{M}=\begin{pmatrix} 1.000\pm0.000 & 0.119\pm0.002 & 0.601\pm0.005 & -0.008\pm0.001 \\ 0.092\pm0.003 & 0.185\pm0.005 & 0.149\pm0.002 & 0.714\pm0.002 \\ 0.540\pm0.002 & 0.196\pm0.001 & 0.929\pm0.001 & -0.176\pm0.001 \\ 0.043\pm0.002 & -0.745\pm0.005 & 0.196\pm0.001 & 0.169\pm0.001 \end{pmatrix}$$

$$\mathbf{M_D}=\begin{pmatrix} 1.000\pm0.000 & 0.119\pm0.002 & 0.601\pm0.005 & -0.008\pm0.001 \\ 0.119\pm0.002 & 0.798\pm0.004 & 0.040\pm0.001 & -0.001\pm0.000 \\ 0.601\pm0.005 & 0.040\pm0.001 & 0.992\pm0.000 & -0.003\pm0.000 \\ -0.008\pm0.001 & -0.001\pm0.000 & -0.003\pm0.000 & 0.790\pm0.004 \end{pmatrix}$$

$$\mathbf{M_R}=\begin{pmatrix} 1.000\pm0.000 & 0.000\pm0.000 & 0.000\pm0.000 & 0.000\pm0.000 \\ 0.000\pm0.000 & 0.247\pm0.004 & 0.164\pm0.002 & 0.955\pm0.001 \\ 0.000\pm0.000 & 0.191\pm0.001 & 0.958\pm0.001 & -0.214\pm0.003 \\ 0.000\pm0.000 & -0.950\pm0.001 & 0.235\pm0.002 & 0.206\pm0.003 \end{pmatrix}$$

$$\mathbf{M_\Delta} = \begin{pmatrix} 1.000 \pm 0.000 & 0.000 \pm 0.000 & 0.000 \pm 0.000 & 0.000 \pm 0.000 \\ -0.022 \pm 0.003 & 0.946 \pm 0.005 & 0.000 \pm 0.003 & 0.007 \pm 0.003 \\ -0.069 \pm 0.006 & 0.000 \pm 0.003 & 1.016 \pm 0.005 & -0.014 \pm 0.002 \\ 0.024 \pm 0.003 & 0.007 \pm 0.003 & -0.014 \pm 0.002 & 0.996 \pm 0.009 \end{pmatrix}$$

G218 first-order transmitted diffraction

$$\mathbf{M} = \begin{pmatrix} 1.000 \pm 0.000 & 0.005 \pm 0.002 & 0.416 \pm 0.002 & -0.027 \pm 0.003 \\ -0.027 \pm 0.006 & 0.577 \pm 0.010 & 0.037 \pm 0.003 & 0.626 \pm 0.001 \\ 0.427 \pm 0.002 & 0.011 \pm 0.004 & 0.947 \pm 0.002 & -0.077 \pm 0.001 \\ 0.007 \pm 0.001 & -0.637 \pm 0.001 & 0.077 \pm 0.004 & 0.549 \pm 0.002 \end{pmatrix}$$

$$\mathbf{M_D} = \begin{pmatrix} 1.000 \pm 0.000 & 0.005 \pm 0.002 & 0.416 \pm 0.002 & -0.027 \pm 0.003 \\ 0.005 \pm 0.002 & 0.909 \pm 0.001 & 0.001 \pm 0.000 & 0.000 \pm 0.000 \\ 0.416 \pm 0.002 & 0.001 \pm 0.000 & 1.000 \pm 0.000 & -0.006 \pm 0.001 \\ -0.027 \pm 0.003 & 0.000 \pm 0.000 & -0.006 \pm 0.001 & 0.909 \pm 0.001 \end{pmatrix}$$

$$\mathbf{M_R} = \begin{pmatrix} 1.000 \pm 0.000 & 0.000 \pm 0.000 & 0.000 \pm 0.000 & 0.000 \pm 0.000 \\ 0.000 \pm 0.000 & 0.667 \pm 0.003 & 0.057 \pm 0.003 & 0.743 \pm 0.003 \\ 0.000 \pm 0.000 & 0.021 \pm 0.005 & 0.995 \pm 0.000 & -0.094 \pm 0.001 \\ 0.000 \pm 0.000 & -0.745 \pm 0.003 & 0.078 \pm 0.004 & 0.663 \pm 0.004 \end{pmatrix}$$

$$\mathbf{M_\Delta} = \begin{pmatrix} 1.000 \pm 0.000 & 0.000 \pm 0.000 & 0.000 \pm 0.000 & 0.000 \pm 0.000 \\ -0.034 \pm 0.003 & 0.937 \pm 0.006 & 0.002 \pm 0.001 & -0.014 \pm 0.004 \\ 0.037 \pm 0.004 & 0.002 \pm 0.001 & 0.935 \pm 0.002 & 0.013 \pm 0.001 \\ -0.008 \pm 0.002 & -0.014 \pm 0.004 & 0.013 \pm 0.001 & 0.929 \pm 0.001 \end{pmatrix}$$

G315 first-order reflected diffraction

$$\mathbf{M} = \begin{pmatrix} 1.000 \pm 0.000 & 0.157 \pm 0.021 & -0137. \pm 0.013 & -0.241 \pm 0.003 \\ 0.278 \pm 0.004 & 0.428 \pm 0.007 & -0.640 \pm 0.021 & -0.037 \pm 0.016 \\ 0.222 \pm 0.011 & 0.235 \pm 0.012 & 0.107 \pm 0.029 & -0.696 \pm 0.003 \\ -0.074 \pm 0.024 & 0.526 \pm 0.007 & 0.453 \pm 0.002 & 0.176 \pm 0.007 \end{pmatrix}$$

$$\mathbf{M_D} = \begin{pmatrix} 1.000 \pm 0.000 & 0.157 \pm 0.021 & -0.137 \pm 0.013 & -0.241 \pm 0.003 \\ 0.157 \pm 0.021 & 0.960 \pm 0.002 & -0.011 \pm 0.001 & -0.019 \pm 0.003 \\ -0.137 \pm 0.013 & -0.011 \pm 0.001 & 0.957 \pm 0.004 & 0.017 \pm 0.002 \\ -0.241 \pm 0.003 & -0.019 \pm 0.003 & 0.017 \pm 0.002 & 0.977 \pm 0.002 \end{pmatrix}$$

$$\mathbf{M_R} = \begin{pmatrix} 1.000 \pm 0.000 & 0.000 \pm 0.000 & 0.000 \pm 0.000 & 0.000 \pm 0.000 \\ 0.000 \pm 0.000 & 0.586 \pm 0.010 & -0.810 \pm 0.007 & 0.034 \pm 0.024 \\ 0.000 \pm 0.000 & 0.252 \pm 0.009 & 0.142 \pm 0.029 & -0.957 \pm 0.004 \\ 0.000 \pm 0.000 & 0.770 \pm 0.005 & 0.569 \pm 0.010 & 0.288 \pm 0.016 \end{pmatrix}$$

$$\mathbf{M}_\Delta = \begin{pmatrix} 1.000 \pm 0.000 & 0.000 \pm 0.000 & 0.000 \pm 0.000 & 0.000 \pm 0.000 \\ 0.126 \pm 0.007 & 0.768 \pm 0.017 & 0.001 \pm 0.007 & -0.042 \pm 0.010 \\ 0.035 \pm 0.010 & 0.001 \pm 0.007 & 0.747 \pm 0.008 & 0.048 \pm 0.012 \\ -0.057 \pm 0.013 & -0.042 \pm 0.010 & 0.048 \pm 0.012 & 0.750 \pm 0.007 \end{pmatrix}$$

G215 first-order reflected diffraction

$$\mathbf{M} = \begin{pmatrix} 1.000 \pm 0.000 & 0.360 \pm 0.012 & -0.015 \pm 0.001 & -0.013 \pm 0.007 \\ 0.143 \pm 0.002 & 0.312 \pm 0.006 & -0.513 \pm 0.002 & 0.447 \pm 0.009 \\ 0.251 \pm 0.008 & 0.346 \pm 0.008 & -0.290 \pm 0.006 & -0.473 \pm 0.004 \\ 0.316 \pm 0.003 & 0.577 \pm 0.010 & 0.367 \pm 0.002 & 0.211 \pm 0.001 \end{pmatrix}$$

$$\mathbf{M}_\mathbf{D} = \begin{pmatrix} 1.000 \pm 0.000 & 0.360 \pm 0.012 & -0.015 \pm 0.001 & -0.013 \pm 0.007 \\ 0.360 \pm 0.012 & 1.000 \pm 0.000 & -0.003 \pm 0.000 & -0.002 \pm 0.001 \\ -0.015 \pm 0.001 & -0.003 \pm 0.000 & 0.933 \pm 0.005 & 0.000 \pm 0.000 \\ -0.013 \pm 0.007 & -0.002 \pm 0.001 & 0.000 \pm 0.000 & 0.933 \pm 0.005 \end{pmatrix}$$

$$\mathbf{M}_\mathbf{R} = \begin{pmatrix} 1.000 \pm 0.000 & 0.000 \pm 0.000 & 0.000 \pm 0.000 & 0.000 \pm 0.000 \\ 0.000 \pm 0.000 & 0.338 \pm 0.008 & -0.716 \pm 0.002 & 0.611 \pm 0.005 \\ 0.000 \pm 0.000 & 0.515 \pm 0.007 & -0.402 \pm 0.011 & -0.757 \pm 0.004 \\ 0.000 \pm 0.000 & 0.787 \pm 0.007 & 0.570 \pm 0.008 & 0.233 \pm 0.006 \end{pmatrix}$$

$$\mathbf{M}_\Delta = \begin{pmatrix} 1.000 \pm 0.000 & 0.000 \pm 0.000 & 0.000 \pm 0.000 & 0.000 \pm 0.000 \\ 0.033 \pm 0.004 & 0.787 \pm 0.006 & 0.012 \pm 0.006 & 0.035 \pm 0.001 \\ 0.133 \pm 0.006 & 0.012 \pm 0.006 & 0.658 \pm 0.005 & -0.060 \pm 0.005 \\ 0.133 \pm 0.008 & 0.035 \pm 0.001 & -0.060 \pm 0.005 & 0.698 \pm 0.011 \end{pmatrix}$$

G218 first-order reflected diffraction

$$\mathbf{M} = \begin{pmatrix} 1.000 \pm 0.000 & 0.059 \pm 0.006 & -0.086 \pm 0.007 & 0.011 \pm 0.001 \\ 0.117 \pm 0.000 & 0.446 \pm 0.004 & -0.199 \pm 0.010 & 0.785 \pm 0.001 \\ 0.100 \pm 0.002 & 0.227 \pm 0.005 & -0.695 \pm 0.003 & -0.313 \pm 0.007 \\ 0.071 \pm 0.005 & 0.642 \pm 0.004 & 0.372 \pm 0.003 & -0.246 \pm 0.001 \end{pmatrix}$$

$$\mathbf{M}_\mathbf{D} = \begin{pmatrix} 1.000 \pm 0.000 & 0.059 \pm 0.006 & -0.086 \pm 0.007 & 0.011 \pm 0.001 \\ 0.059 \pm 0.006 & 0.996 \pm 0.001 & -0.003 \pm 0.000 & 0.000 \pm 0.000 \\ -0.086 \pm 0.007 & -0.003 \pm 0.000 & 0.998 \pm 0.000 & 0.000 \pm 0.000 \\ 0.011 \pm 0.001 & 0.000 \pm 0.000 & 0.000 \pm 0.000 & 0.995 \pm 0.000 \end{pmatrix}$$

$$\mathbf{M}_\mathbf{R} = \begin{pmatrix} 1.000 \pm 0.000 & 0.000 \pm 0.000 & 0.000 \pm 0.000 & 0.000 \pm 0.000 \\ 0.000 \pm 0.000 & 0.474 \pm 0.003 & -0.221 \pm 0.007 & 0.852 \pm 0.004 \\ 0.000 \pm 0.000 & 0.315 \pm 0.002 & -0.861 \pm 0.005 & -0.399 \pm 0.010 \\ 0.000 \pm 0.000 & 0.822 \pm 0.003 & 0.458 \pm 0.006 & -0.339 \pm 0.002 \end{pmatrix}$$

$$\mathbf{M}_{\Delta} = \begin{pmatrix} 1.000 \pm 0.000 & 0.000 \pm 0.000 & 0.000 \pm 0.000 & 0.000 \pm 0.000 \\ 0.066 \pm 0.003 & 0.925 \pm 0.002 & -0.010 \pm 0.003 & 0.009 \pm 0.002 \\ 0.031 \pm 0.002 & -0.010 \pm 0.003 & 0.793 \pm 0.003 & -0.026 \pm 0.002 \\ 0.069 \pm 0.001 & 0.009 \pm 0.002 & -0.026 \pm 0.002 & 0.786 \pm 0.002 \end{pmatrix}$$
